# Supplementary material for: Burden in caregivers of children with congenital Zika syndrome in Pernambuco, Brazil: analysis and application of the Zarit burden interview scale
Source: PeerJ. 2023 Feb 2;11:e14807. doi: 10.7717/peerj.14807 (PMC9899425; doi:10.7717/peerj.14807)
Supplement: Supplemental Information 1 [file peerj-11-14807-s001.docx]

**INSTRUMENT II**

**2. Sociodemographic profile of the responsible caregiver:**

Responsible for home care for children with microcephaly caused by the Zika virus:

A. Gender:

1. Male ( )

2. Female ( )

3. Uninformed ( )

B. Degree of kinship:

1. Father ( )

2. Mother ( )

3.Other _____________________________________________________________

C. Age in years: _________________________

D. Marital status:

1. Married ( )

2. Single ( )

3. Divorced ( )

4. Widower ( )

E. Number of living children: _______________________

F. Numbers of children with microcephaly: ____________________

G. In which gestational period “mother case” did Zika virus disease occur:

1. In the first quarter ( )

2. No segundo trimestre ( )

3. In the third quarter ( )

4. Don't know how to inform ( )

H. Family income:

In reais: __________________________________________________________

I. Do you have any medical treatment after taking care of the child.

1. Yes ( )

2. No ( )

If yes, what treatment do you do?

___________________________________________________________________

J. Do you work.

1. Yes ( )

2. No ( )

If the alternative is Yes. What is your profession?

___________________________________________________________________

L. How long have you been this child's caregiver in months?

___________________________________________________________________

M. How much time do you dedicate per day for the child in hours?

___________________________________________________________________

N. Telephone for contact with area code: optional item.

O. Activities as a caregiver: DO YOU PARTICIPATE?

_ Child feeding;

1. Yes ( )

2. No ( )

_Child medication;

1. Yes ( )

2. No ( )

_Child bath

1. Yes ( )

2. No ( )

_Dress or help dress the child;

1. Yes ( )

2. No ( )

_You help transport the child.

1. Yes ( )

2. No ( )

P. Did you receive any guidance regarding the dilution and administration of these indicated medications?

1. Yes ( )

2. No ( )

If the answer is “yes”, which professional gave you the information?

___________________________________________________________________

Q. What are the difficulties described at the time of dilution and administration of medications:

___________________________________________________________________

R. What are the difficulties in swallowing presented by children at the time of medication administration:

__________________________________________________________________

S. The child has been hospitalized for choking at the time of swallowing for food or medication:

1. Yes ( )

2. No ( )

If the answer is yes, talk about what caused the choking and if there was a need for medical attention:

___________________________________________________________________

T. What are the main difficulties encountered by you in caring for the child?

___________________________________________________________________

U. Given the difficulties presented, how do you think you could improve them?

___________________________________________________________________
