# Supplementary material for: Burden in caregivers of children with congenital Zika syndrome in Pernambuco, Brazil: analysis and application of the Zarit burden interview scale
Source: PeerJ. 2023 Feb 2;11:e14807. doi: 10.7717/peerj.14807 (PMC9899425; doi:10.7717/peerj.14807)
Supplement: Supplemental Information 2 [file peerj-11-14807-s002.docx]

**INSTRUMENTO II**

**2. Perfil sociodemográfico do cuidador responsável:**

Responsável pelos cuidados no domicílio as crianças com microcefalia pelo vírus zika:

A. Sexo:

1. Masculino ( )

2. Feminino ( )

3. Não informado ( )

B. Grau de parentesco:

1. Pai ( )

2. Mãe ( )

3.Outro _____________________________________________________________

C. Idade em Anos: _________________________

D. Estado civil:

1. Casado (a) ( )

2. Solteiro (a) ( )

3. Divorciado (a) ( )

4. Viúvo (a) ( )

E. Números de filhos vivos: _______________________

F. Números de filhos com microcefalia: ____________________

G. Em que período gestacional “caso mãe” ocorreu à doença por vírus zika:

1. No primeiro trimestre ( )

2. No segundo trimestre ( )

3. No terceiro trimestre ( )

4. Não sabe informar ( )

H. Renda Familiar:

Em Reais: __________________________________________________________

I. Você faz algum tratamento médico depois que passou a cuidar da criança.

1. Sim ( )

2. Não ( )

Em caso de sim qual tratamento que você faz?

___________________________________________________________________

J. Você trabalha.

1. Sim ( )

2. Não ( )

Em caso da alternativa ser Sim. Qual sua profissão?

___________________________________________________________________

L. Quanto tempo você é cuidador desta criança em meses?

___________________________________________________________________

M. Quanto tempo você dedica por dia para a criança responda em horas?

___________________________________________________________________

N. Telefone para contato com DDD: item opcional.

O. Atividades como cuidador: **É VOCÊ QUE PARTICIPA?**

_ Alimentação da criança;

1. Sim ( )

2. Não ( )

_Medicação da criança;

1. Sim ( )

2. Não ( )

_Banho da criança;

1. Sim ( )

2. Não ( )

_Veste ou ajuda a vestir a criança;

1. Sim ( )

2. Não ( )

_Você auxilia no transporte da criança.

1. Sim ( )

2. Não ( )

P. você recebeu alguma orientação enquanto à diluição e administração dos destes medicamentos indicados.

1. Sim ( )

2. Não ( )

Em caso da resposta ser afirmativa “sim” qual (is) profissional (is) lhe deu (deram) as informações?

___________________________________________________________________

Q. Quais as dificuldades a descrita no momento da diluição e administração dos medicamentos:

___________________________________________________________________

R. Quais as dificuldades na deglutição apresentados pelas crianças no momento da administração dos medicamentos:

__________________________________________________________________

S. Já houve internamento por engasgo na hora da deglutição por alimento ou medicação:

1. Sim ( )

2. Não ( )

Em caso da resposta ser afirmativa fale sobre o que causou o engasgo e se houve necessidade de atendimento médico:

___________________________________________________________________

T. Qual (is) principal (is) dificuldade (s) encontrada (s) por você no cuidado com a criança?

___________________________________________________________________

U. Diante das dificuldades apresentadas como você acha poderia melhora-las?

___________________________________________________________________
