# Supplementary material for: Burden in caregivers of children with congenital Zika syndrome in Pernambuco, Brazil: analysis and application of the Zarit burden interview scale
Source: PeerJ. 2023 Feb 2;11:e14807. doi: 10.7717/peerj.14807 (PMC9899425; doi:10.7717/peerj.14807)
Supplement: Supplemental Information 3 [file peerj-11-14807-s003.docx]

**FACULDADE DE MEDICINA DO ABC**

**PROGRAMA DE PÓS-GRADUAÇÃO EM CIÊNCIAS DA SAÚDE**

**Research Project: “Impact of care overload and the main difficulties encountered by caregivers, according to the comorbidities presented in children with microcephaly caused by the Zika virus”.** The Zarit scale (Zarit Burden Interview). It presents 22 questions that assess the impact of care in the spheres; physical, psychological and social, whose score can vary from 0-4.45 The sum of these questions is 88 points and a cut-off point was established to define the caregiver's burden. It should be marked according to the degree of intensity in which the burden of care occurs: **0** corresponds to **Never**, **1** corresponds to **Rarely**, **2** corresponds to **Sometimes**, **3** corresponds to **Often** and **4** corresponds to **Always**.

**ZARIT SCALE (Zarit Burden Interview)**

| 1. Do you feel that THE CHILD asks for more help than he/she needs? | 0 | 1 | 2 | 3 | 4 |
| --- | --- | --- | --- | --- | --- |
| 2. Do you feel that because of the time you spend with THE CHILD you don't have enough time for yourself? |  |  |  |  |  |
| 3. Do you feel stressed between taking care of the CHILD and your other responsibilities with family and work? |  |  |  |  |  |
| 4. Do you feel ashamed of the CHILD's behavior? |  |  |  |  |  |
| 5. Do you feel irritated when THE CHILD is around? |  |  |  |  |  |
| 6. Do you feel that THE CHILD negatively affects your relationships with other family members or friends? |  |  |  |  |  |
| 7. Do you feel fear for the CHILD's future? |  |  |  |  |  |
| 8. Do you feel that THE CHILD depends on you? |  |  |  |  |  |
| 9. Do you feel tense when THE CHILD is around? |  |  |  |  |  |
| 10. Do you feel that your health has been affected because of your involvement with THE CHILD? |  |  |  |  |  |
| 11. Do you feel that you do not have as much privacy as you would like because of the CHILD? |  |  |  |  |  |
| 12. Do you feel that your social life has been harmed as a result of having to take care of the CHILD? |  |  |  |  |  |
| 13. Do you not feel comfortable having visitors at home because of the CHILD? |  |  |  |  |  |
| 14. Do you feel that THE CHILD expects you to take care of them as if you were the only person they can depend on? |  |  |  |  |  |
| 15. Do you feel that you do not have enough money to take care of the CHILD in addition to your other expenses? |  |  |  |  |  |
| 16. Do you feel that you will be unable to take care of the CHILD for much longer? |  |  |  |  |  |
| 17. Do you feel that you have lost control of your life since the CHILD's illness? |  |  |  |  |  |
| 18. Would you like to let someone else take care of the CHILD? |  |  |  |  |  |
| 19. Do you feel in doubt about what to do for the CHILD? |  |  |  |  |  |
| 20. Do you feel that you should be doing more for the CHILD? |  |  |  |  |  |
| 21. Do you feel that you could take better care of your CHILD? |  |  |  |  |  |
| 22. In general, how much do you feel burdened by taking care of the CHILD? |  |  |  |  |  |
