# Supplementary material for: Burden in caregivers of children with congenital Zika syndrome in Pernambuco, Brazil: analysis and application of the Zarit burden interview scale
Source: PeerJ. 2023 Feb 2;11:e14807. doi: 10.7717/peerj.14807 (PMC9899425; doi:10.7717/peerj.14807)
Supplement: Supplemental Information 4 [file peerj-11-14807-s004.docx]

**FACULDADE DE MEDICINA DO ABC**

**PROGRAMA DE PÓS-GRADUAÇÃO EM CIÊNCIAS DA SAÚDE**

**Projeto de Pesquisa**: **“Impacto da sobrecarga de cuidados e as principais dificuldades encontradas pelos cuidadores, de acordo com as comorbidades apresentadas em crianças com microcefalia pelo vírus zika”.** A escala Zarit (Zarit Burden Interview). Apresenta 22 questões que avaliam o impacto do cuidado nas esferas; física, psicológica e social, cuja pontuação pode variar de 0-4.45 O somatório destas questões é de 88 pontos e foi estabelecido um ponto de corte para definir a sobrecarga do cuidador. Deve ser assinalada de acordo com grau de intensidade em que ocorre a sobrecarga do cuidado em: **0** corresponde a **Nunca**, **1** corresponde a **Raramente**, **2** corresponde a **Algumas vezes**, **3** corresponde a **Frequentemente** e **4** corresponde a **Sempre**.

**ESCALA DE ZARIT (Zarit Burden Interview)**

| 1. O (a) senhor (a) sente que **A CRIANÇA** pede mais ajuda do que ele (a) necessita? | 0 | 1 | 2 | 3 | 4 |
| --- | --- | --- | --- | --- | --- |
| 2. O (a) senhor (a) sente que por causa do tempo que gasta com **A CRIANÇA** não tem tempo suficiente pra si mesmo? |  |  |  |  |  |
| 3. O (a) senhor (a) se sente estressado (a) entre cuidar **da CRIANÇA** e suas outras responsabilidades com a família e o trabalho? |  |  |  |  |  |
| 4. O (a) senhor (a) se sente envergonhado (a) com o comportamento **da CRIANÇA**? |  |  |  |  |  |
| 5. O (a) senhor (a) se sente irritado (a) quando **A CRIANÇA** está por perto? |  |  |  |  |  |
| 6. O (a) senhor (a) sente que **A CRIANÇA** afeta negativamente seus relacionamentos com outros membros da família ou amigos? |  |  |  |  |  |
| 7. O (a) senhor (a) sente receio pelo futuro **da** **CRIANÇA**? |  |  |  |  |  |
| 8. O (a) senhor (a) sente que **A CRIANÇA** depende do senhor (a)? |  |  |  |  |  |
| 9. O (a) senhor (a) se sente tenso quando **A CRIANÇA** está por perto? |  |  |  |  |  |
| 10. O (a) senhor (a) sente que a sua saúde foi afetada por causa do seu envolvimento com **A CRIANÇA**? |  |  |  |  |  |
| 11. O (a) senhor (a) sente que não tem tanta privacidade como gostaria por causa da **CRIANÇA**? |  |  |  |  |  |
| 12. O (a) senhor (a) sente que sua vida social tem sido prejudicada em razão de ter de cuidar da **CRIANÇA?** |  |  |  |  |  |
| 13. O (a) senhor (a) não se sente a vontade para receber visitas em casa por causa **da** **CRIANÇA**? |  |  |  |  |  |
| 14. O (a) senhor (a) sente que **A CRIANÇA** espera que o (a) senhor (a) cuide dela como se fosse à única pessoa de quem ela pode depender? |  |  |  |  |  |
| 15. O (a) senhor (a) sente que não tem dinheiro suficiente para cuidar **da** **CRIANÇA** somando-se as suas outras despesas? |  |  |  |  |  |
| 16. O (a) senhor (a) sente que será incapaz de cuidar **da CRIANÇA** por muito mais tempo? |  |  |  |  |  |
| 17. O (a) senhor (a) sente que perdeu o controle da sua vida desde a doença **da** **CRIANÇA**? |  |  |  |  |  |
| 18. O (a) senhor (a) gostaria de simplesmente deixar que outra pessoa cuidasse **da CRIANÇA**? |  |  |  |  |  |
| 19. O (a) senhor (a) se sente em dúvida sobre o que fazer **pela CRIANÇA**? |  |  |  |  |  |
| 20. O (a) senhor (a) sente que deveria estar fazendo mais **pela CRIANÇA**? |  |  |  |  |  |
| 21. O (a) senhor (a) sente que poderia cuidar melhor **da CRIANÇA**? |  |  |  |  |  |
| 22. De uma maneira geral, quanto o (a) senhor se sente sobrecarregado (a) por cuidar **da CRIANÇA**? |  |  |  |  |  |
